# Smoking enhanced the expression of c-kit in chromophobe renal cell carcinoma

**DOI:** 10.18332/tid/170432

**Published:** 2023-10-06

**Authors:** Jiahao Jiang, Lanxin Yang, Mingzhu Chen, Fei Xiao, Yan Zeng, Hengcheng Zhu, Yanqin Li, Lingqi Liu

**Affiliations:** 1 Department of Urology, Renmin Hospital, Wuhan University, Wuhan, China; 2 School of Pharmaceutical Sciences, Wuhan University, Wuhan, China

**Keywords:** TCGA, smoking, c-kit, chromophobe renal cell carcinoma

## Abstract

**INTRODUCTION:**

Smoking is an important risk factor for inducing renal cell carcinoma (RCC), but its specific mechanism affecting the development of RCC remains to be elucidated. Chromophobe RCC (ChRCC) is a subtype of RCC. Many studies have shown smoking is closely associated with RCC occurrence and c-kit plays a critical role in the progression of RCC, however, few studies focus on ChRCC. This study investigated the molecular mechanism between smoking and the c-kit pathway in ChRCC.

**METHODS:**

Differentially expressed genes (DEGs) were obtained from The Cancer Genome Atlas (TCGA) in ChRCC and the expression of KIT in ChRCC was analyzed through the TCGA database combined with Gene Expression Omnibus (GEO) and oncomine databases. Moreover, Gene Ontology (GO) and Kyoto Encyclopedia of Genes and Genomes (KEGG) pathway analyses and Protein Protein Interaction (PPI) network analysis were performed to explore the function of KIT and correlated DEGs as well as its co-expression genes in ChRCC. Finally, ChRCC patient samples were used to verify the effect of smoking on the c-kit expression.

**RESULTS:**

The results showed that KIT is one of the DEGs and plays a vital role in ChRCC tumorigenesis. Interestingly, the expression of c-kit in cancer tissues of 27 smoking patients was significantly higher than that of 25 non-smoking patients (p<0.05), which suggests smoking might enhance the expression of c-kit in ChRCC patients.

**CONCLUSIONS:**

Our results demonstrate that smoking might play a pivotal role in the ChRCC tumorigenesis via a pathway related to c-kit, and provided new insight into the relationship between smoking and the c-kit pathway in ChRCC.

## INTRODUCTION

Renal cell carcinoma (RCC) is a highly malignant cancer with high incidence, ranking fifth in the world in incidence, accounting for 2–3% of all malignant tumors in adults^1^. In China, the prevalence of RCC ranks second in the urinary system, and the mortality rate is the first in urinary system tumors^2^. RCC originates from renal tubular epithelial cells and the most common type of RCC is clear cell carcinoma with an incidence of 70–75%. Chromophobe RCC (ChRCC) is the second most frequent subtype of non-clear cell renal carcinoma and accounts for approximately 5–8% of RCC and has a better prognosis than the other subtypes of RCC such as clear cell, papillary and collecting duct^3^. Because of the lack of apparent clinical symptoms, many patients initially diagnosed with RCC are in the advanced stages, with approximately 1/3 patients presenting with regional or distant metastases^4^, But patients with ChRCC usually present at an earlier stage and only 1.8–4.9% have metastatic disease at diagnosis^5^. For localized and re-sectable ChRCC, surgery is the gold standard treatment. However, for advanced or metastatic ChRCC, so far, no strong evidence has been presented with regard to the ideal first-line treatment^3^. Therefore, the underlying mechanisms of ChRCC should be further explored for the therapy and prognosis of patients.

KIT is a receptor belonging to the type III receptor tyrosine kinase family, which is involved in hematopoiesis, melanogenesis, and gametogenesis. Mutations in c-kit can lead to dysregulation of its activity and affect the normal physiological activities of cells. These mutations cause damage to special cell types, including macrocytic anemia, infertility, and loss of skin pigmentation^6^. Mutation of c-kit are implicated in the pathogenesis of various neoplasms, including gastrointestinal stromal tumor (GIST), systemic mastocytosis, acute myelogenous leukemia, and germ cell tumors. Many studies have shown that c-kit plays a very important role in the occurrence and development of different cancers^7^. The overexpression of c-kit can promote the occurrence and development of KIT-dependent cell types of tumors, such as gastrointestinal stromal tumors, mast cell tumors, and germ cell tumors^8^. For gastrointestinal stromal tumors, leukemias and other tumors with abnormally high expression of c-kit, drugs that downregulate the expression of c-kit have already been available and have good clinical effects.

Smoking is a known risk factor for RCC^9^. The risk of RCC is positively correlated with the duration and the cumulative amount of smoking^10^. Nicotine, the main ingredient in tobacco, is identified to facilitate tumorigenesis and accelerate metastasis in tumor. Nicotine is also an addictive ingredient and could produce psychoactive effects^11^. Besides, it is an important factor that enables smokers to maintain smoking behavior^12^.

In this study, we aimed to explore the role of KIT in ChRCC via comprehensive bioinformatic analysis and investigate the role of smoking in the ChRCC tumorigenesis which might occur through a pathway related to the hub gene c-kit.

## METHODS

### The Cancer Genome Atlas (TCGA) database

The RNASeq expression data of ChRCC was downloaded through TCGA *biolinks* Rpackage. The *tidyverse* and *magrittr* Rpackages were used to organize the expression matrix of ChRCC expression data, and *biomaRt* Rpackage was applied to annotate the genes in the expression matrix^13^.

### Gene Expression Omnibus (GEO) database

The GEO Platform files of GSE(GEO Series)15641 and GSE11151 were obtained from the GEO microarray series, and were searched through the ID of KIT for the expression data of KIT in ChRCC kidney tissues^14^.

### Identification of DEGs (differentially expressed genes)

The above expression matrix was visualized with *DESeq2* Rpackage, and differentially expressed genes were screened. The standard of data selection was log Fold change >1, p<0.05. According to this criterion, the genes that are differentially expressed in ChRCC were screened out from the data. STRING database and Cytoscape were used to analyze KIT and DEGs as PPI networks^15^.

### Functional enrichment analysis

*Corrplot* Rpackage was used to screen differentially expressed genes that have a strong correlation with KIT in ChRCC. Correlation coefficient ≥0.4 and false discovery rate (FDR) <0.05 were set as the standard. GO (Gene Ontology) and KEGG (Kyoto Encyclopedia of Genes and Genomes) pathways were used to perform the functional annotation of DEGs. GO analysis was done for assessment of biological processes (BP), molecular functions (MF), and cellular components (CC). KEGG (https://www.kegg.jp/) contains 18 databases with genome information and can understand the functions of cells and organisms from genome sequences and other molecular data sets. GO and KEGG pathway analyses results were processed by the *clusterprofiler*, *enrichplot*, and *ggplot2* packages with a p-value and q-value of 0.05 as the cut-offs. The GSEA (gene set enrichment analysis) soft was used to analyze the enrichment results of hub genes^16^.

### Co-expressed genes analysis

The co-expressed genes of KIT in ChRCC were analyzed and visualized through the Linkedomics database. The co-expressed genes were screened, and the criteria were p<0.05 and correlation coefficient ≥0.4. Metascape was used for GO and KEGG pathways to analysis those co-express genes^10^.

### PPI (Protein-Protein Interaction) network construction

The PPI network of DEGs with KIT correlation coefficient ≥0.7 in ChRCC was constructed using the STRING database.

### Clinical sample

A total of 52 male ChRCC patients were included from Renmin Hospital of Wuhan University and First hospital of Peking University between 2003 and 2018. All subjects signed an informed consent form. This study was approved by the human research ethics committee, school of pharmaceutical sciences, Wuhan University (No.20183060001). The screening conditions of the clinical samples were as follows: ChRCC, of which 27 cases had a history of smoking and 25 cases had no history of smoking. For patients with a history of smoking, relevant information was obtained through medical records and telephone return visits. Formalin-fixed, paraffin-embedded samples were retrieved from the archives of the department of pathology at Renmin Hospital of Wuhan University.

### Immunohistochemistry (IHC)

The unstained paraffin sections of tissue from ChRCC patients were collected and analyzed by immunohistochemistry. Briefly, the patients’ sections were incubated with c-kit mouse monoclonal antibody (1:150, Origene Technologies, #TA801036) overnight at 4^o^C. Then, tissue sections were washed with PBS three times and labeled with a HRP-conjugated Goat anti-Rabbit IgG H&L antibody (Abcam, #ab205718) at a dilution of 1:1000 for 2 h at room temperature. After IHC staining was completed, pictures (400×) were taken with an Olympus inverted fluorescence microscope, and Image-J was used for quantitative analysis.

### Statistical analysis

RNA-seq data downloaded from TCGA were analyzed for differences using the DESeq package, and differences between the two groups of data were detected by Student’s t-test. The relationship between KIT expression levels and DEGs was analyzed by Spearman rank correlation. Data were expressed as mean ± SD and analyzed by ANOVA (analysis of variance) with the appropriate between- and within-subjects factors for the different experiments. A p<0.05 was considered statistically significant.

## RESULTS

### Identification of KIT as an overexpressed gene in ChRCC

**Figure 1:**
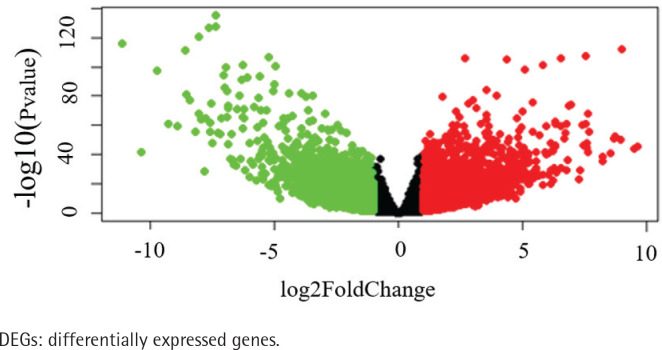
Identification of KIT as an overexpressed gene in ChRCC. Identification of DEGs in ChRCC. Volcano plot of all DEGs. The red dots represent up-regulated genes, the green dots represent downer-gulated genes, the black dots represent unchanged genes

**Figure 2:**
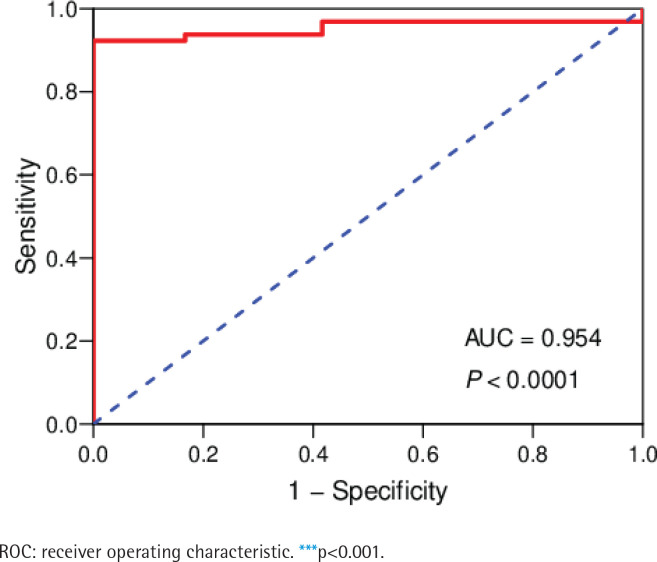
ROC curve analysis of KIT in ChRCC

The data of 65 ChRCC samples and 24 healthy kidney tissue samples were extracted from the TCGA database. PCA analysis was performed and revealed that the collection of 65 ChRCC and 24 normal samples were positively different, which confirmed the gene expression profile difference between tumor and normal tissues in ChRCC (Supplementary file Figure 1). Subsequently, the DESeq2 Rpackage was used to screen differentially expressed genes (DEGs) between ChRCC and adjacent tissues (Figure 1). A total of 1980 DEGs were screened and the screening criteria were p<0.05, |log2 fold-change| ≥2 and FDA <0.05. The results are presented in Figure 1, which showed 985 up-regulated genes including KIT and 995 down-regulated genes. ROC curve test was performed on 65 ChRCC samples, the area under the curve was 0.954 (95% CI: 0.9062–0.9990), which was statistically significant (p<0.0001), indicating that KIT was meaningful for the diagnosis of ChRCC (Figure 2).

Next, we analyzed the correlation of KIT and DEGs by using Corrplot Rpackage in ChRCC, the results are shown in Supplementary file Figure 2. The screening criteria were p<0.05 and KIT-related DEGs were screened with COR >0.6 as the criterion. Furthermore, the current study used the STRING tool to explore protein-protein interaction (PPI) networks and illuminate the interplay among KIT and its co-expressed genes. The results of the PPI revealed the interaction between DEGs and KIT in ChRCC (Supplementary file Figure 3). Moreover, we explored the potential biological functions of KIT and its related DEGs in ChRCC by performing GO and KEGG pathway analyses through Metascape. In Supplementary file Figure 4, it shown that in ChRCC the GO enrichment analysis involved in KIT-related genes mainly includes circulatory system process, renal system process, regulation of body fluid levels, enzyme linked receptor protein signaling pathway, positive regulation of secretion, and ion homeostasis. To further investigate the function of KIT in ChRCC, DisGeNET was used to analyze the relationship between KIT and human disease-related genes and mutations. The abnormal expression of KIT and its correlated DEGs might be a signal related to kidney failure disease, smoking behaviors, renal tubular acidosis, distal, autosomal recessive, pseudohypoaldosteronism, type II and hyperactive renin-angiotensin system disease (Supplementary file Figure 4).

### KIT expression in different cancers

**Figure 3:**
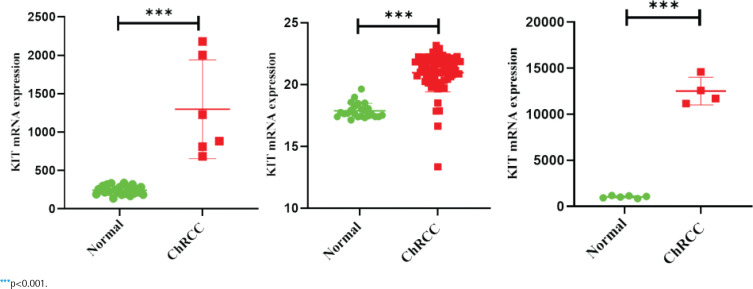
The mRNA expression level of KIT between tumor and normal samples in ChRCC patients on TCGA and GEO database, data sets GSE15641 and GSE11151

GEPIA and Oncomine databases were used to analyze and compare the differential expression of KIT in different tumors (Supplementary file Figure 5). In the two sets of different data, KIT showed abnormally high expression level in ChRCC. Next, GEO and TCGA databases were used to analyze the differential expression of KIT in adjacent tissues and ChRCC. KIT was significantly up-regulated expression in 65 ChRCC samples compared with 24 healthy kidney tissue samples. The GSE15641 and GSE11151 in the GEO database include 23 healthy kidney tissues and 6 ChRCC samples, 4 ChRCC tissues and 6 adjacent tissues, respectively. The expression level of KIT was positively increased in ChRCC (Figure 3).

### KEGG pathway of KIT in ChRCC

The expression data set of KIT in ChRCC was used for GSEA analysis. Taking the expression data of KIT in ChRCC as the grouping standard, the ChRCC expression data set was analyzed by GSEA_4.1.0 to obtain the KEGG pathway of KIT in ChRCC. GSEA results indicated that numerous typical tumor-related signaling pathways were significantly enriched in ChRCC patients, such as oxidative phosphorylation, PPAR signaling pathway, Notch signaling pathway, P53 signaling pathway, and primary immunodeficiency (Supplementary file Figure 6).

### Functional enrichment analysis of KIT and its co-expressed genes

Based on the TCGA database, we analyzed the co-expressed genes of KIT in ChRCC. As shown in Supplementary file Figure 7, 42 genes were positively correlated with KIT in ChRCC. Conversely, 31 gene expressions were negatively correlated with KIT (FDA<0.01, p<0.05). From the heat map, the 50 most significant genes that were positively or negatively correlated with KIT can be seen (Supplementary file Figure 7). To further explore the potential function of KIT and its co-expressed genes in ChRCC tumorigenesis, the GO and KEGG pathway analyses were performed. For GO analysis, in terms of the biological process, cellular components and molecular functions, the KIT and its co-expressed genes in ChRCC were significantly enriched in kinase binding, regulation of cellular response to stress, positive regulation of transferase activity, cellular response to hormone stimulus, positive regulation of cell death, transmembrane receptor protein tyrosine kinase signaling , gland development, protein kinase activity, epithelial cell differentiation, response to inorganic substance, and epithelial cell proliferation (Supplementary file Figure 8). Among the KEGG pathways, EGFR tyrosine kinase inhibitor resistance, ErbB signaling pathway, MAPK signaling pathway and calcium signaling pathway were involved in ChRCC tumorigenesis (Supplementary file Figure 9). The enrichment network diagram of related genes showed that the enrichment network includes PI3K-Akt signaling pathway, EGFR tyrosine kinase inhibitor resistance, TGF-beta signaling pathway, senescence and autophagy in cancer and cytokine signaling in immune system (Supplementary file Figure 10).

### Smoking can induce the c-kit expression in ChRCC patient samples

**Figure 4:**
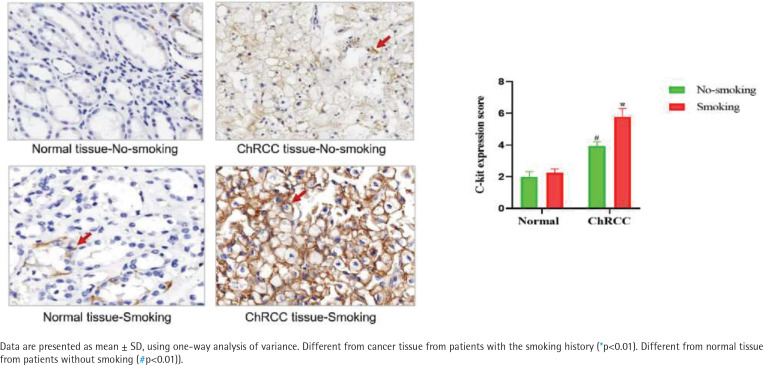
Smoking induced the c-kit expression in ChRCC patients. Immunohistochemical detection of the expression of c-kit in ChRCC patients’ samples (400×). Quantification of the expression of c-kit in ChRCC patients’ renal tissues

According to the regulations on the use of biological samples in China, data on 52 ChRCC male patients after surgery were collected from Renmin Hospital of Wuhan University and First hospital of Peking University, including 27 patients with smoking history and 25 patients without smoking history. In ChRCC, the expression of c-kit in cancer tissues of smoking patients was significantly higher than that of non-smoking patients [F (1,11)=53.19, p<0.05] (Figure 4).

## DISCUSSION

RCC is one of the most common urinary system malignancies with high morbidity and high mortality globally. ChRCC is relatively rare and biologically distinct when compared to ccRCC. In this study, we identified the up-regulated gene KIT in ChRCC through the comparison of gene expression profiles based on the TCGA datasets. Subsequently, functional and pathway enrichment analyses were performed to find potential biological function of KIT and its related genes. In addition, smoking has a significant activation on the expression of c-kit in ChRCC patients. Thus, these results suggested the involvement of c-kit in the tumorigenesis and progression of ChRCC, and smoking might play a pivotal role in the ChRCC tumorigenesis via a pathway related to c-kit.

After comparing the ChRCC samples and normal kidney tissue samples, we identified 985 up-regulated genes including KIT and 995 down-regulated genes. GO and KEGG pathways analyses demonstrated that these differentially expressed genes (DEGs) are involved in ion homeostasis and regulation of ion transport. In renal physiology, electrolyte and water homeostasis is facilitated by ion transport mechanisms. Ion homeostasis and regulation of ion transport play a vital role in cell survival. Previous studies found that down-regulated pathways in the kidney tumor cells included ion homeostasis^17^. Gamper et al.^18^ also reported that a large number of membrane transport proteins including ion transport proteins were sensitive to the phosphoinositides in the plasma membrane. According to the KEGG pathway analysis results of KIT, we found that KIT was significantly associated with oxidative phosphorylation, fatty acid metabolism, peroxisome proliferator-activated receptors (PPARs) signaling pathway, adipocytokine signaling pathway, glutathione metabolism, Notch signaling pathway, P53 signaling pathway and primary immunodeficiency. In the previous studies, SETD2 deficiency induced a metabolic switch toward enhanced oxidative phosphorylation in ccRCC, which can be related to peroxisome proliferator-activated receptor γcoactivator-1 (PGC1α)-mediated metabolic networks^19^. Besides, Xu et al.^20^ showed the potential roles of Acyl-CoA Thioesterase 8 in the regulation of oxidative phosphorylation. Apart from oxidative phosphorylation, Notch signaling also plays a crucial role in cancer stem-like cells maintaining stemness and mediating chemotaxia in RCC and blocking of Notch signaling resulted in attenuation of proliferation and restrained the growth of ccRCC cell lines^21^. Accumulating evidence has revealed significant correlation between the P53 signaling pathway and tumorigenesis and prognosis in many tumor types^22^. Although several studies indicated that KIT is a crucial inducement for oncogenesis, the potential molecular mechanism between KIT and ChRCC still remains unclear. Thus, these findings indicate that KIT might regulate oncogenesis of ChRCC via mediating oxidative phosphorylation, Notch signaling pathway and P53 signaling pathway.

Based on these findings, we next explored the potential function of KIT co-expression genes in ChRCC. Seventy-three genes were significantly correlated with KIT. These analyses were followed by GO functions and KEGG pathway analyses to clarify the potential molecular mechanisms. As expected, these genes are mainly associated with EGFR tyrosine kinase inhibitor resistance, ErbB signaling pathway, MAPK signaling pathway, and so on. In fact, EGFR/ Akt signaling pathway plays an irreplaceable role in the tumorigenesis and progression, and is involved in tumor cell proliferation and invasion^23^. Furthermore, it is worth mentioning that MAPK signaling pathway was reported to play an essential role in the tumorigenesis^24^. Much evidence has demonstrated that special proteins could regulate cell proliferation and invasion by activating MAPK signaling pathway in RCC^25^. Fan et al.^26^ reported Kallikrein-related peptidase 4 accelerated ChRCC progression via ERK/ AKT signaling pathway. A PPI network demonstrated that PI3K-Akt signaling pathway was significantly enriched in our study. Previous studies revealed that PI3K/AKT/mTOR pathway was associated with cell proliferation and migration in RCC^27^. These data indicate that our results based on the TCGA database have significant value and also confirmed previous reports on the pivotal role of KIT and its associated genes in ChRCC tumorigenesis and disease progression.

A large number of epidemiological reports have characterized the association between smoking and RCC and it is also known to be in a dose-dependent manner^28-31^. To further confirm the potential role of smoking in ChRCC tumorigenesis, the expression of c-kit was tested in ChRCC patients with or without smoking history. Previous studies revealed that according to a retrospective investigation, smoking seems to be a risk factor associated to ccRCC and pRCC, but not for the ChRCC subtype^28^. In contrast, another study has investigated the relationship between RCC histological subtypes and risk factors and showed no subtype differences for association with smoking^32^. Similarly, our data exhibited that the ChRCC patients with smoking history had significantly higher c-kit expression level. Several studies revealed c-kit expression and KIT mutation in renal tumors^33^. For example, Zhou et al.^34^ found the KIT oncogene specifically upregulated on the cell membranes of ChRCC and indicated KIT could be a new therapeutic target in ChRCC. Amin et al.^35^ revealed that KIT could be a useful immunophenotypic marker for ChRCC, but the therapeutic value is uncertain because of the lack of mutations. Zimpfer et al.^33^ proved that c-kit expression was found in the majority of ChRCC, but without usual c-kit activating mutations. Abbosh et al.^36^ reported c-kit overexpression in 67% metastatic ChRCC samples. These findings suggest that the upregulation of the c-kit might be of great diagnostic and therapeutic value in ChRCC. Since smoking could induce c-kit expression in ChRCC patients, our results indicate that the role of smoking in the oncogenesis of ChRCC might happen through a pathway related to c-kit.

## CONCLUSIONS

We identified the overexpression gene KIT and explored the potential function of KIT and its associated genes in ChRCC through integrated bioinformatics analysis based on the TCGA database. In addition, it was found that nicotine could induce the c-kit pathway in clinical samples. Our study is first to demonstrate, to our knowledge, a link between smoking, c-kit expression and ChRCC tumorigenesis. However, the limited number of ChRCC patients in this study should be considered, and further studies are still need to explore the special molecular mechanism of smoking and the c-kit pathway in ChRCC.

## Data Availability

All data include in this study are available upon request by contact with the corresponding author.

## References

[1] Sung H, Ferlay J, Siegel RL (2021). Global Cancer Statistics 2020: GLOBOCAN estimates of incidence and mortality worldwide for 36 cancers in 185 countries. CA Cancer J Clin.

[2] Chen W, Zheng R, Baade PD (2016). Cancer statistics in China, 2015. CA Cancer J Clin.

[3] Garje R, Elhag D, Yasin HA, Acharya L, Vaena D, Dahmoush L (2021). Comprehensive review of chromophobe renal cell carcinoma. Crit Rev Oncol Hematol.

[4] Choueiri TK, Motzer RJ (2017). Systemic therapy for metastatic renal-cell carcinoma. N Engl J Med.

[5] Przybycin CG, Cronin AM, Darvishian F (2011). Chromophobe renal cell carcinoma: a clinicopathologic study of 203 tumors in 200 patients with primary resection at a single institution. Am J Surg Pathol.

[6] Heldin CH, Lennartsson J (2013). Structural and functional properties of platelet-derived growth factor and stem cell factor receptors. Cold Spring Harb Perspect Biol.

[7] Sheikh E, Tran T, Vranic S, Levy A, Bonfil RD (2022). Role and significance of c-KIT receptor tyrosine kinase in cancer: a review. Bosn J Basic Med Sci.

[8] O'Neill TW, Löhr CV (2021). Mast cell tumors and histiocytomas in domestic goats and diagnostic utility of CD117/c-kit and Iba1 immunohistochemistry. Vet Pathol.

[9] Wei JH, Feng ZH, Cao Y (2019). Predictive value of single-nucleotide polymorphism signature for recurrence in localised renal cell carcinoma: a retrospective analysis and multicentre validation study. Lancet Oncol.

[10] Zhou Y, Zhou B, Pache L (2019). Metascape provides a biologist-oriented resource for the analysis of systems-level datasets. Nat Commun.

[11] Fowler CD, Turner JR, Imad Damaj M (2020). Molecular Mechanisms Associated with Nicotine Pharmacology and Dependence. Handb Exp Pharmacol.

[12] Jain G, Jaimes EA (2013). Nicotine signaling and progression of chronic kidney disease in smokers. Biochem Pharmacol.

[13] Wang Z, Jensen MA, Zenklusen JC (2016). A Practical Guide to The Cancer Genome Atlas (TCGA). Methods Mol Biol.

[14] Clough E, Barrett T (2016). The Gene Expression Omnibus Database. Methods Mol Biol.

[15] Doncheva NT, Morris JH, Gorodkin J, Jensen LJ (2019). Cytoscape StringApp: network analysis and visualization of proteomics data. J Proteome Res.

[16] Canzler S, Hackermüller J (2020). multiGSEA: a GSEA-based pathway enrichment analysis for multi-omics data. BMC Bioinformatics.

[17] Boer JM, Huber WK, Sültmann H (2001). Identification and classification of differentially expressed genes in renal cell carcinoma by expression profiling on a global human 31,500-element cDNA array. Genome Res.

[18] Gamper N, Shapiro MS (2007). Regulation of ion transport proteins by membrane phosphoinositides. Nat Rev Neurosci.

[19] Liu J, Hanavan PD, Kras K (2019). Loss of SETD2 induces a metabolic switch in renal cell carcinoma cell lines toward enhanced oxidative phosphorylation. J Proteome Res.

[20] Xu CL, Chen L, Li D, Chen FT, Sha ML, Shao Y (2020). Acyl-CoA Thioesterase 8 and 11 as novel biomarkers for clear cell renal cell carcinoma. Front Genet.

[21] Xiao W, Gao Z, Duan Y, Yuan W, Ke Y (2017). Notch signaling plays a crucial role in cancer stem-like cells maintaining stemness and mediating chemotaxis in renal cell carcinoma. J Exp Clin Cancer Res.

[22] Liu Q, Fang Q, Ji S, Han Z, Cheng W, Zhang H (2018). Resveratrol-mediated apoptosis in renal cell carcinoma via the p53/AMP-activated protein kinase/mammalian target of rapamycin autophagy signaling pathway. Mol Med Rep.

[23] Li M, Cai L, Wang X (2021). RHBDD1 promotes proliferation, migration, invasion and EMT in renal cell carcinoma via the EGFR/AKT signaling pathway. Mol Med Rep.

[24] Chen Z, Zhang Y, Wu X (2021). Gαi1 promoted proliferation, migration and invasion via activating the Akt-mTOR/Erk-MAPK signaling pathway in renal cell carcinoma. Onco Targets Ther.

[25] Hong B, Zhou J, Ma K (2019). TRIB3 promotes the proliferation and invasion of renal cell carcinoma cells via activating MAPK signaling pathway. Int J Biol Sci.

[26] Fan B, Niu Y, Zhang A (2022). KLK4 silencing inhibits the growth of chromophobe renal cell carcinoma through ERK/AKT signaling pathway. Kidney Blood Press Res.

[27] Xie J, Lin W, Huang L (2018). Bufalin suppresses the proliferation and metastasis of renal cell carcinoma by inhibiting the PI3K/Akt/mTOR signaling pathway. Oncol Lett.

[28] Patel NH, Attwood KM, Hanzly M (2015). Comparative analysis of smoking as a risk factor among renal cell carcinoma histological subtypes. J Urol.

[29] Chow WH, Dong LM, Devesa SS (2010). Epidemiology and risk factors for kidney cancer. Nat Rev Urol.

[30] Capitanio U, Bensalah K, Bex A (2019). Epidemiology of renal cell carcinoma. Eur Urol.

[31] Padala SA, Barsouk A, Thandra KC (2020). Epidemiology of renal cell carcinoma. World J Oncol.

[32] Purdue MP, Moore LE, Merino MJ (2013). An investigation of risk factors for renal cell carcinoma by histologic subtype in two case-control studies. Int J Cancer.

[33] Zimpfer A, Janke S, Hühns M (2014). C-kit overexpression is not associated with KIT gene mutations in chromophobe renal cell carcinoma or renal oncocytoma. Pathol Res Pract.

[34] Santarpia L, Lippman SM, El-Naggar AK (2012). Targeting the MAPK-RAS-RAF signaling pathway in cancer therapy. Expert Opin Ther Targets.

[35] Amin J, Xu B, Badkhshan S (2018). Identification and validation of radiographic enhancement for reliable differentiation of CD117(+) benign renal oncocytoma and chromophobe renal cell carcinoma. Clin Cancer Res.

[36] Abbosh P, Sundararajan S, Millis SZ (2018). Molecular and genomic profiling to identify actionable targets in chromophobe renal cell cancer. Eur Urol Focus.

