# Supplementary material for: Smoking enhanced the expression of c-kit in chromophobe renal cell carcinoma
Source: Tob Induc Dis. 2023 Oct 6;21:126. doi: 10.18332/tid/170432 (PMC10557055; doi:10.18332/tid/170432)
Supplement: Supplementary file 1 [file TID-21-126-s1.pdf]

Supplementary figure 1 PCA analysis of ChRCC cancer samples and normal samples in the TCGA cohort

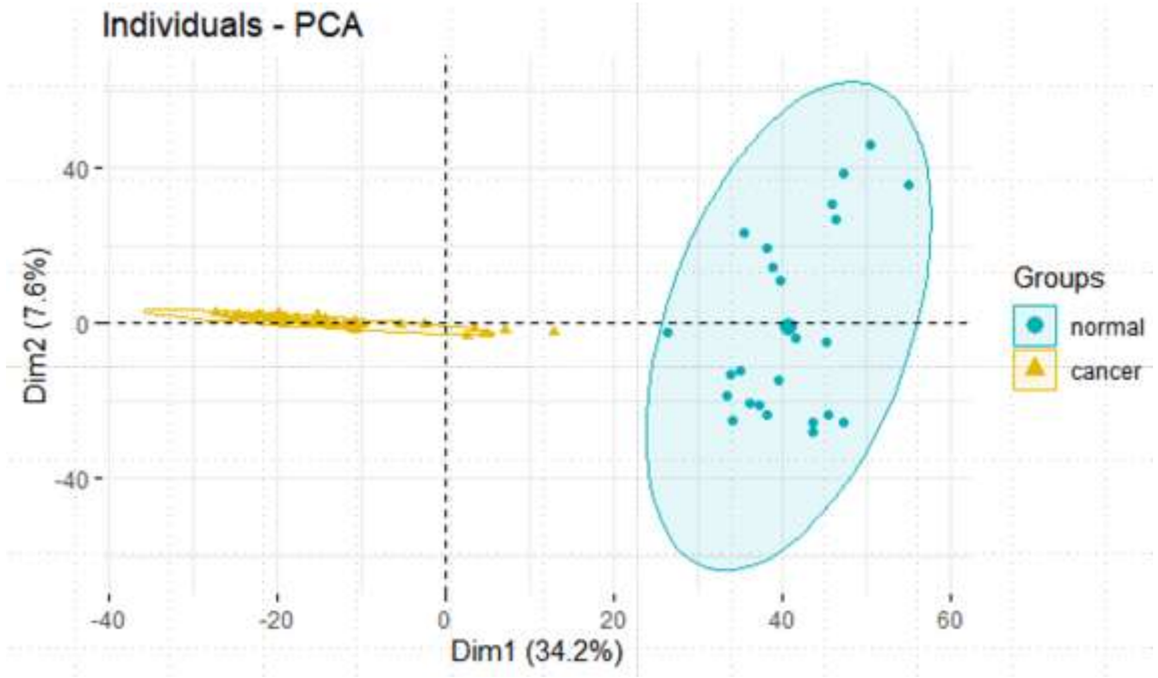

Supplementary figure 2 KIT-correlated genes in ChRCC. Corrlation index ≥0.4， $P<0.05$

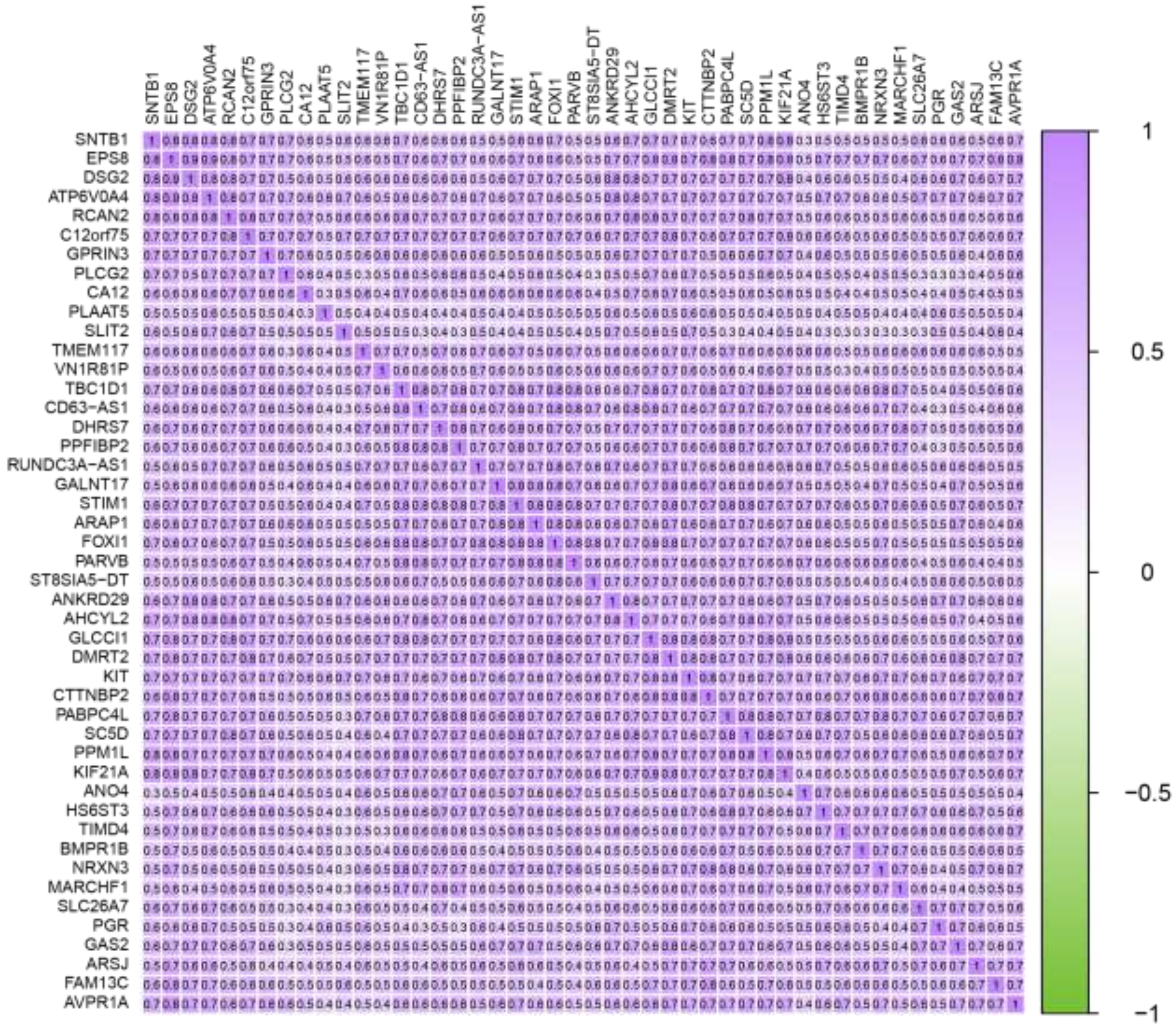

Supplementary figure 3 PPI networks of KIT and its correlated DEGs.

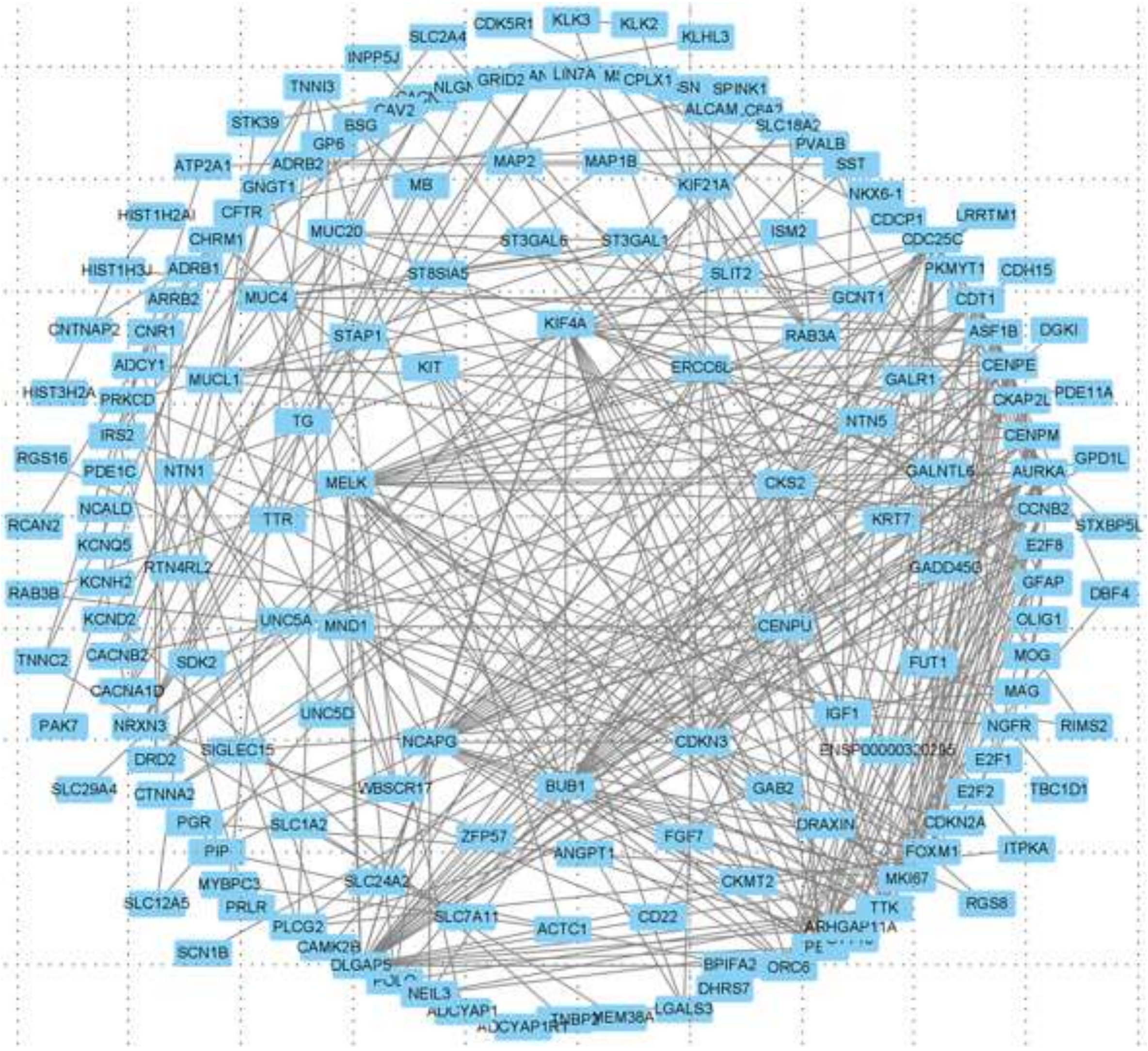

Summary Statistics	
Number of nodes	166
Number of edges	411
Avg. number of neighbors	4.952
Network diameter	14
Network radius	7
Characteristic path length	6.296
Clustering coefficient	0.303
Network density	0.030
Network heterogeneity	1.206
Network centralization	0.135
Connected components	1
Analysis time (sec)	0.027

Supplementary figure 4 GO and KEGG enrichment pathway analysis based on the DEGs and summary of enrichment analysis in DisGeNET.

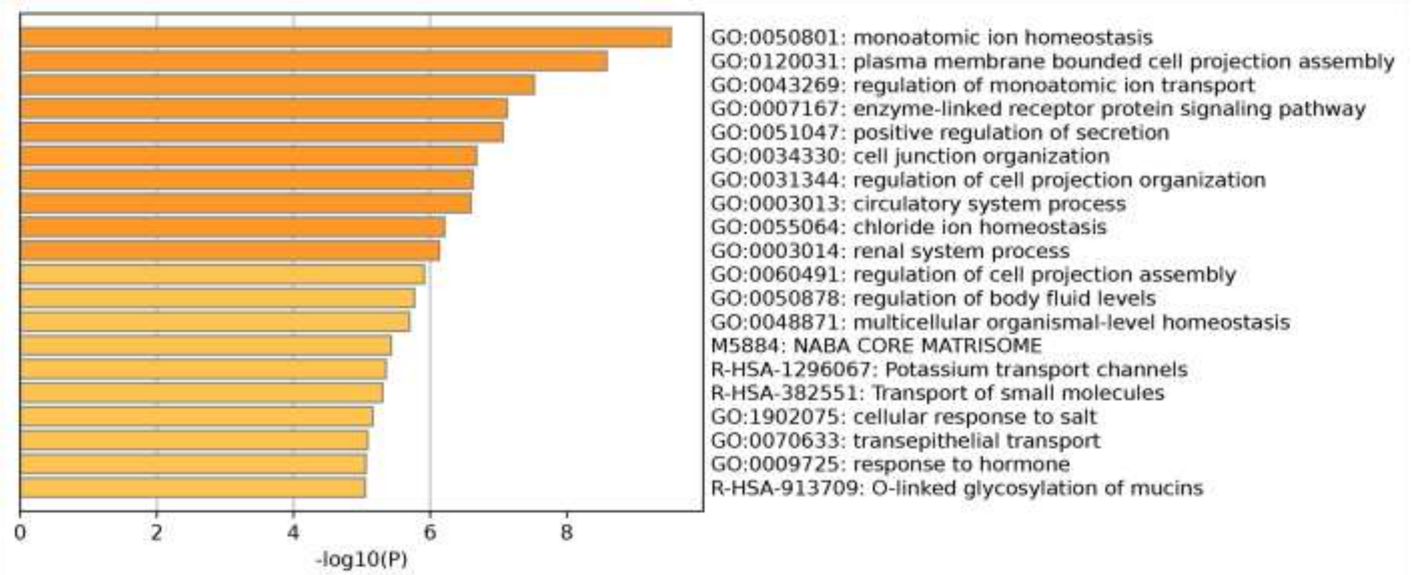

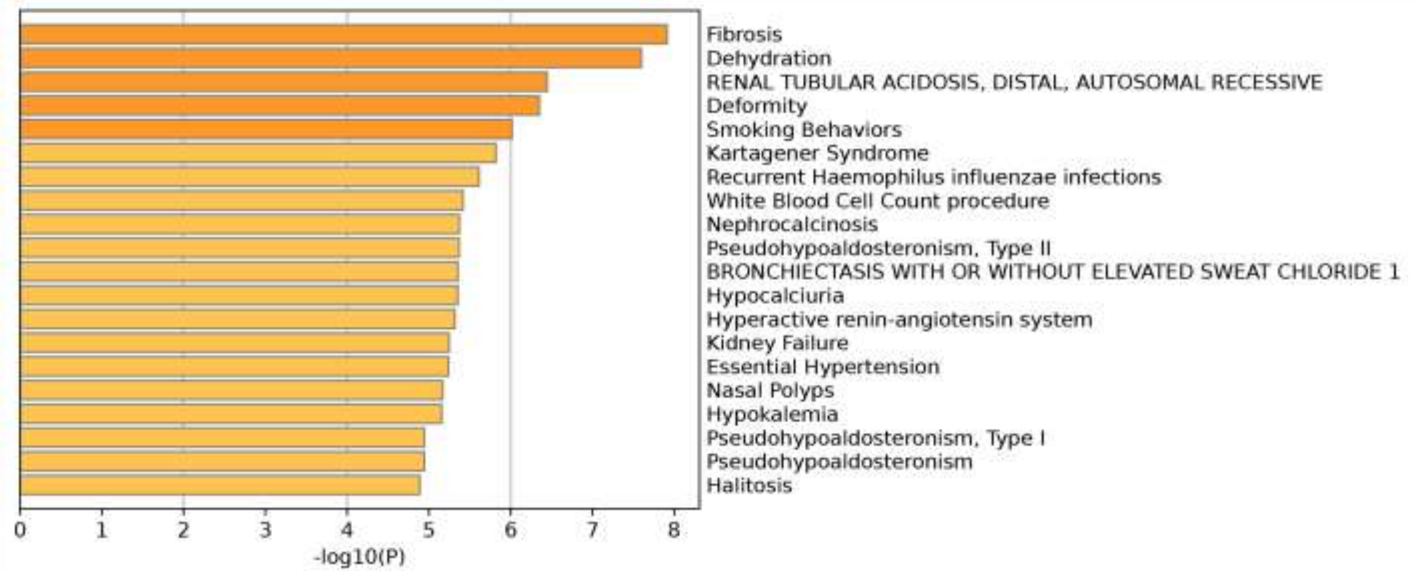

Supplementary figure 5 Differential expression of KIT in cancer tissues and adjacent tissues in GEPIA database.

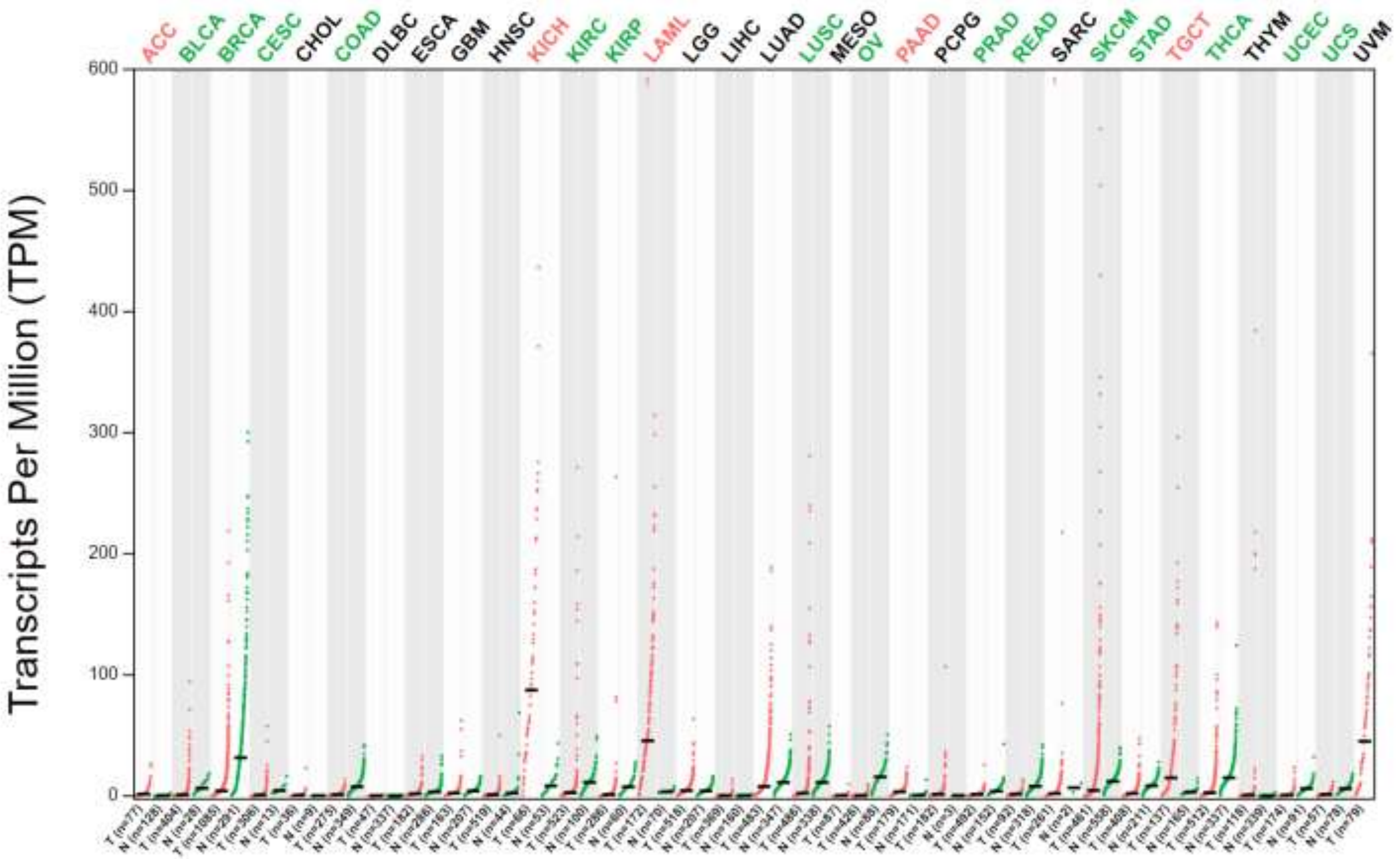

Supplementary Figure 6 KEGG pathway of KIT in ChRCC. GSEA analysis in the TCGA cohort. The result of GESA showed that high KIT expression samples were enriched into Notch and P53 signaling pathway, etc. GSEA: Gene Set Enrichment Analysis.

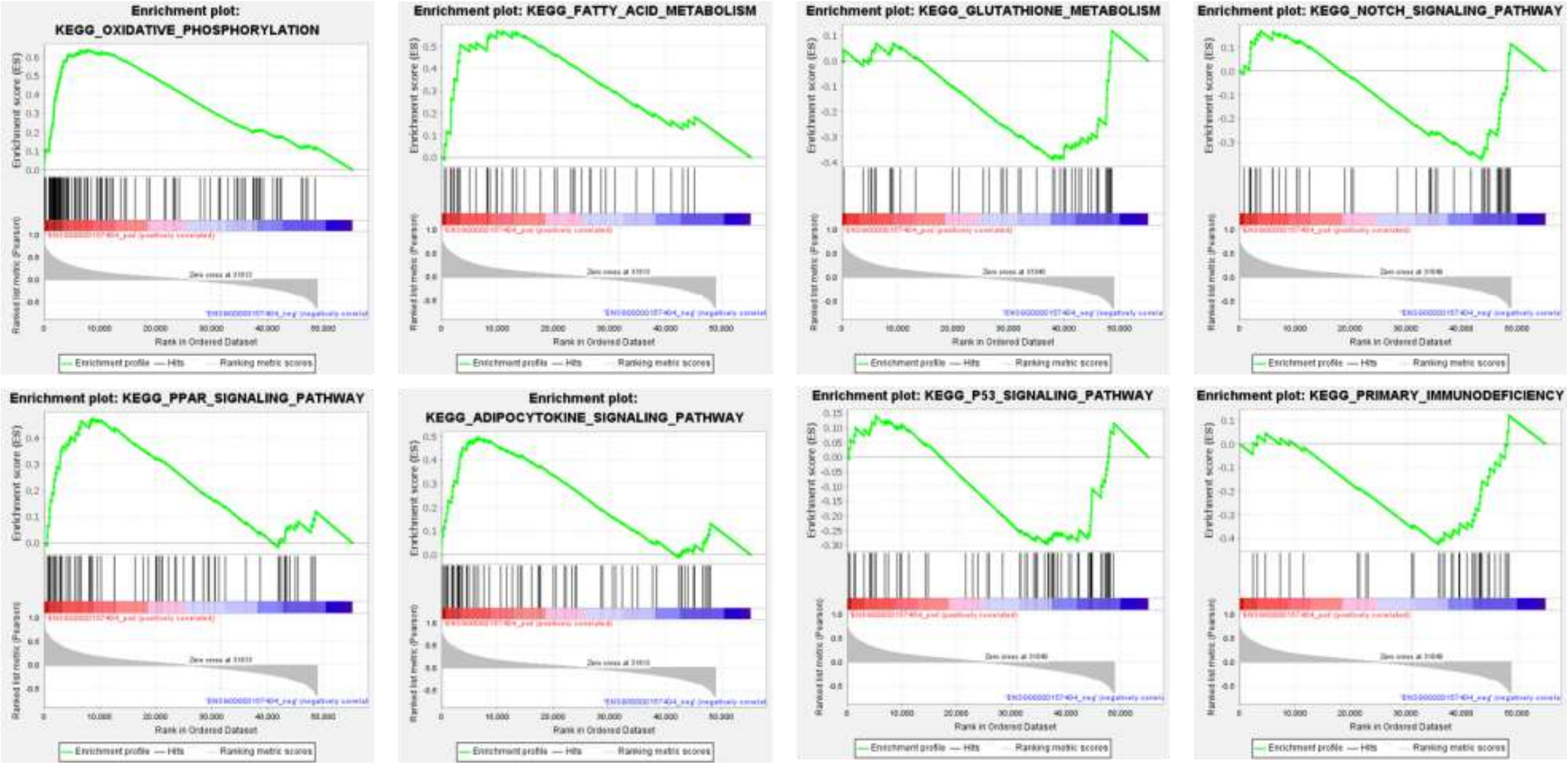

Supplementary Figure 7 Functional enrichment analysis of KIT and its co-expressed Genes. All KIT-related genes, the first 50 genes positively related to KIT and the first 50 genes negatively related to KIT.

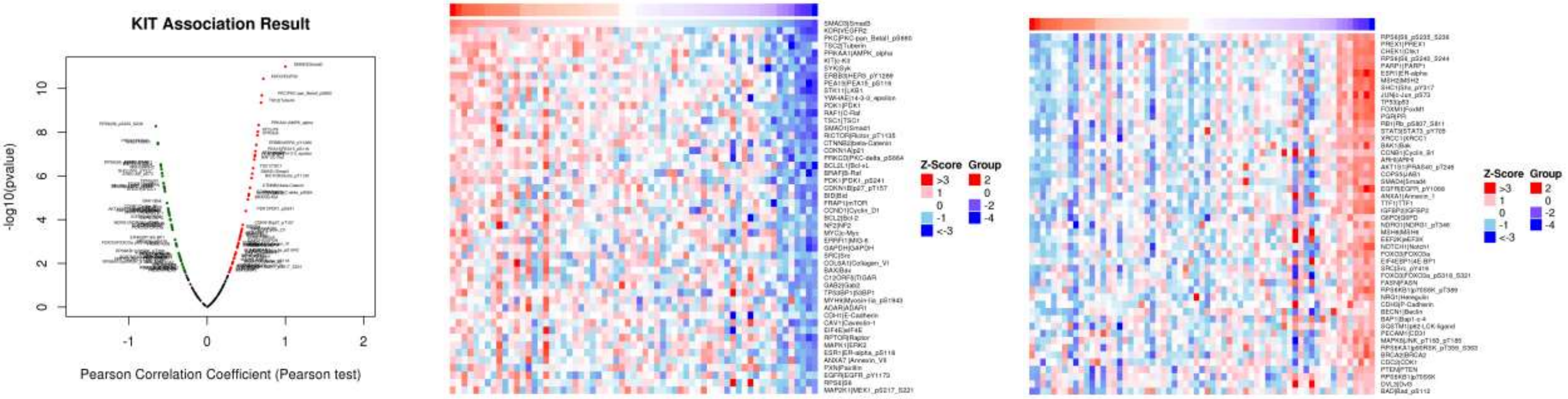

Supplementary figure 8 The GO function of KIT co-expression gene enriched.

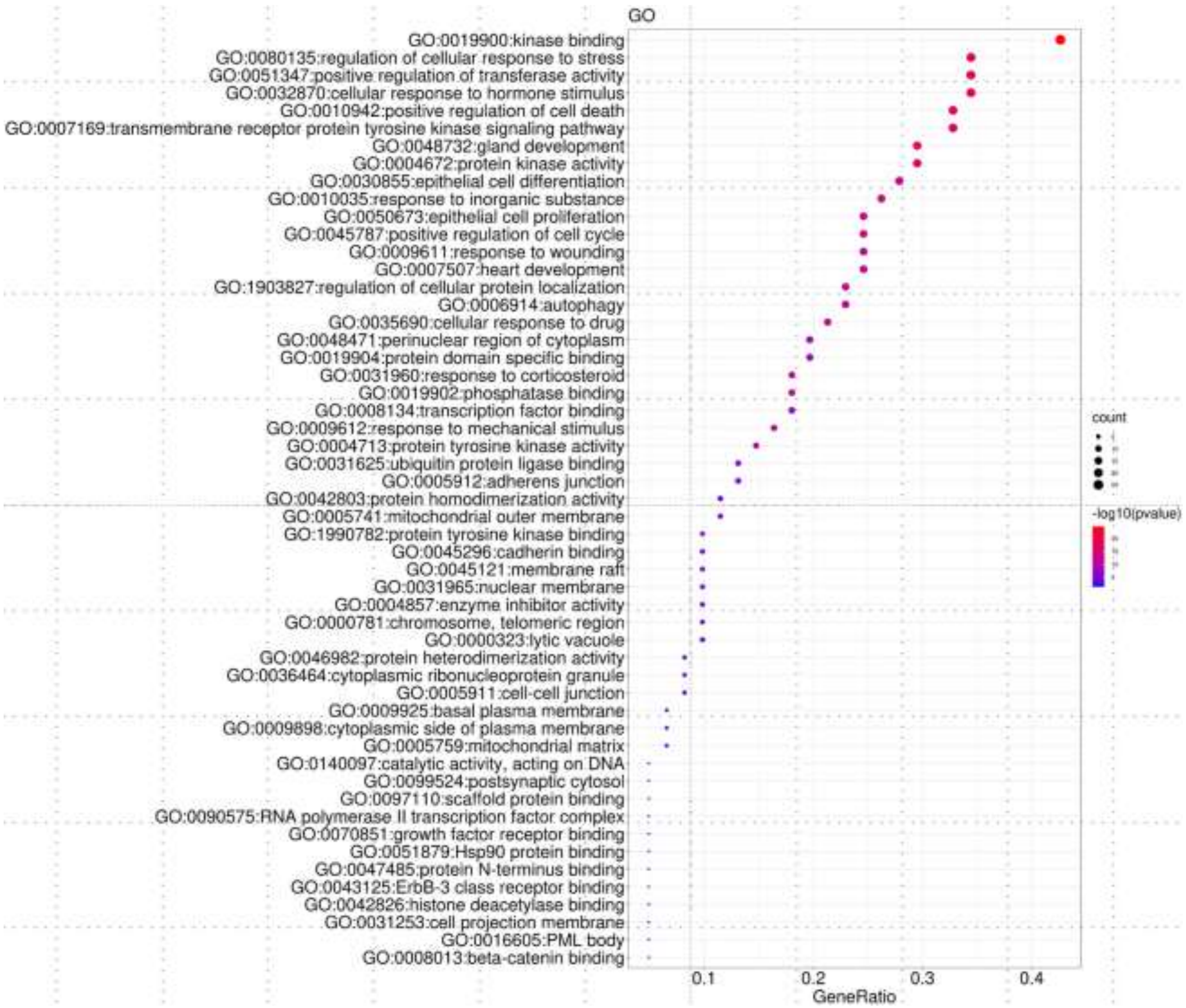

Supplementary figure 9   The KEGG pathway of KIT co-expression gene.

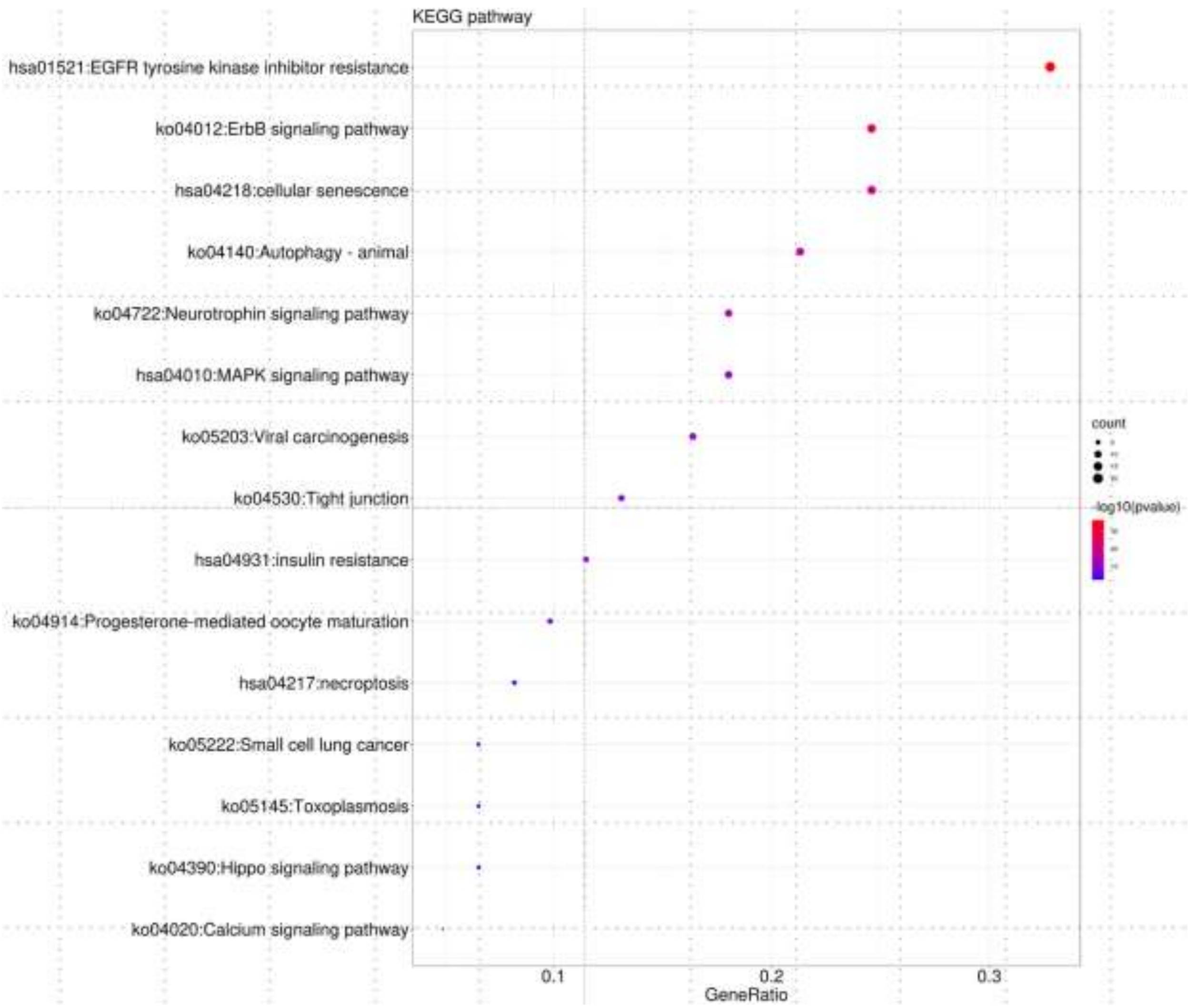

Supplementary figure 10   The enrichment network diagram of related genes

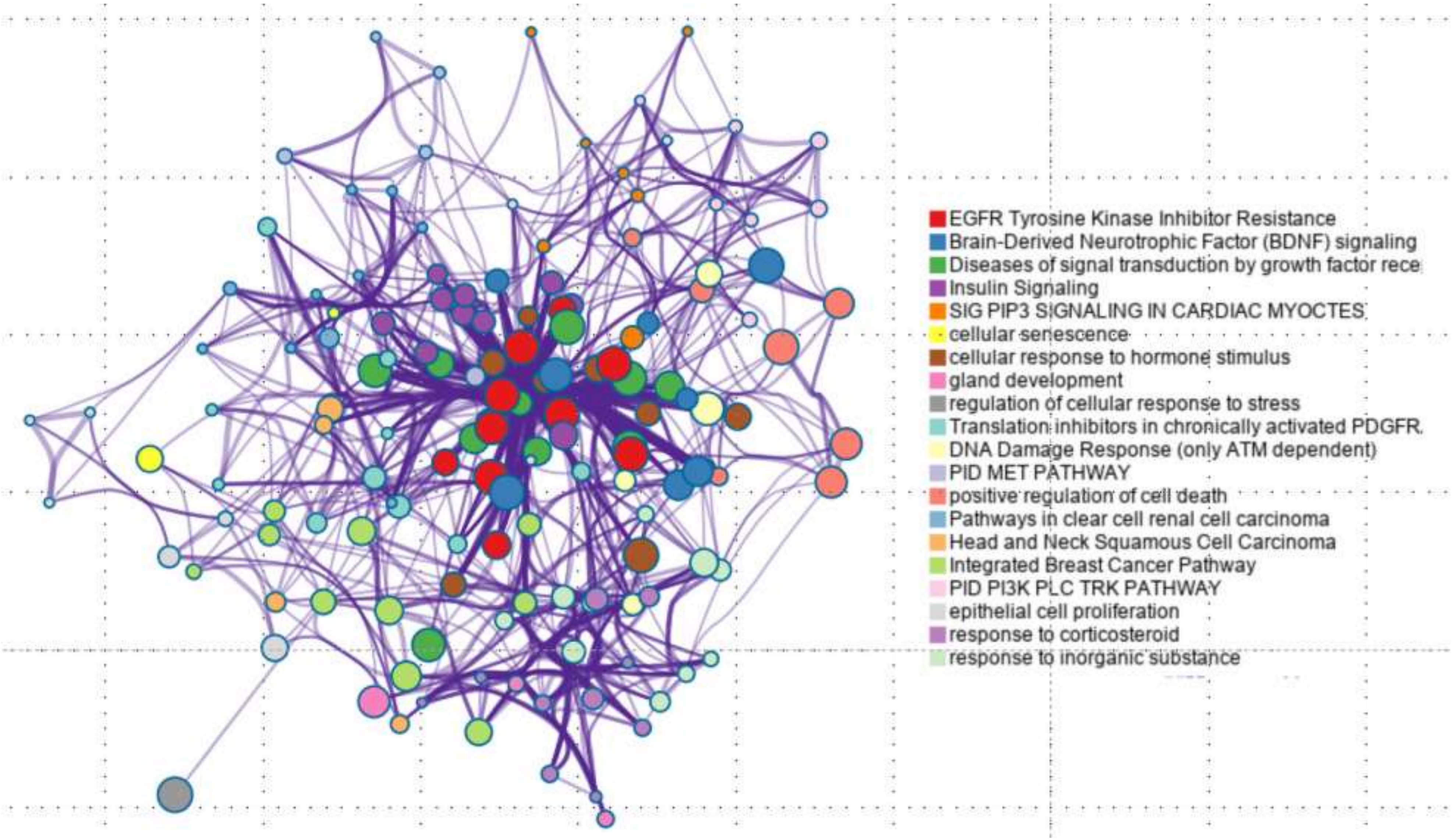

© 2023 Jiang J. et al.
